# A Randomized Trial of Vitamin D_3_ Supplementation in Children: Dose-Response Effects on Vitamin D Metabolites and Calcium Absorption

**DOI:** 10.1210/jc.2013-2728

**Published:** 2013-10-03

**Authors:** R. D. Lewis, E. M. Laing, K. M. Hill Gallant, D. B. Hall, G. P. McCabe, D. B. Hausman, B. R. Martin, S. J. Warden, M. Peacock, C. M. Weaver

**Affiliations:** The University of Georgia (R.D.L., E.M.L., D.B.Hal., D.B.Hau.), Athens, Georgia 30602; Purdue University (K.M.H.G., G.P.M., B.R.M., C.M.W.), West Lafayette, Indiana 47907; and Indiana University (K.M.H.G., S.J.W., M.P.), Indianapolis, Indiana 42602

## Abstract

**Context:**

Changes in serum vitamin D metabolites and calcium absorption with varying doses of oral vitamin D_3_ in healthy children are unknown.

**Objective:**

Our objective was to examine the dose-response effects of supplemental vitamin D_3_ on serum vitamin D metabolites and calcium absorption in children living at two U.S. latitudes.

**Design:**

Black and white children (n = 323) participated in a multisite (U.S. latitudes 34° N and 40° N), triple-masked trial. Children were randomized to receive oral vitamin D_3_ (0, 400, 1000, 2000, and 4000 IU/d) and were sampled over 12 weeks in winter. Serum 25-hydroxyvitamin D (25(OH)D) and 1,25-dihydroxyvitamin D (1,25(OH)_2_D) were measured using RIA and intact PTH (iPTH) by immunoradiometric assay. Fractional calcium absorption was determined from an oral stable isotope ^44^Ca (5 mg) in a 150-mg calcium meal. Nonlinear and linear regression models were fit for vitamin D metabolites, iPTH, and calcium absorption.

**Results:**

The mean baseline 25(OH)D value for the entire sample was 70.0 nmol/L. Increases in 25(OH)D depended on dose with 12-week changes ranging from −10 nmol/L for placebo to 76 nmol/L for 4000 IU. Larger 25(OH)D gains were observed for whites vs blacks at the highest dose (*P* < .01). Gains for 1,25(OH)_2_D were not significant (*P* = .07), and decreases in iPTH were not dose-dependent. There was no dose effect of vitamin D on fractional calcium absorption when adjusted for pill compliance, race, sex, or baseline 25(OH)D.

**Conclusion:**

Large increases in serum 25(OH)D with vitamin D_3_ supplementation did not increase calcium absorption in healthy children living at 2 different latitudes. Supplementation with 400 IU/d was sufficient to maintain wintertime 25(OH)D concentrations in healthy black, but not white, children.

In the absence of sufficient nonskeletal outcomes data (1–3), the 2010 Institute of Medicine (IOM) committee considered only bone studies to estimate pediatric vitamin D requirements (4). Although the committee concluded that there were no skeletal benefits of 25-hydroxyvitamin D (25(OH)D) >50 nmol/L and that intakes of 600 IU vitamin D per day were satisfactory, knowledge gaps persist. For example, 25(OH)D responds to oral vitamin D in a dose-dependent manner between 200 and 2000 IU/d in children (5–8); however, 25(OH)D responses to higher intakes are unknown. Most childhood intervention trials lack 1,25-dihydroxyvitamin D (1,25(OH)_2_D) measures, but those that report 1,25(OH)_2_D show dose-dependent increases (9). It remains unknown whether 1,25(OH)_2_D responds to vitamin D intakes >2000 IU, whether increases in 1,25(OH)_2_D are related to increased calcium absorption, or whether the 1,25(OH)_2_D response is similar in white and nonwhite populations.

Controversy exists regarding a 25(OH)D inflection point in children at which maximal suppression of serum intact PTH (iPTH) occurs and calcium absorption is maximized. Although childhood trials identified iPTH inflection points of ∼75 nmol/L 25(OH)D (5, 10), we were unable to reproduce similar results (11). Child intervention studies suggest that iPTH suppression occurs with higher vitamin D intake (8), although doses <2000 IU/d do not suppress iPTH in blacks (6). Based on these data, considering iPTH suppression for defining optimal 25(OH)D in children remains questionable. We have shown that blacks vs whites have greater calcium retention (12), but relationships between calcium absorption and 25(OH)D have not been detected in cross-sectional child studies (13). Likewise, supplementation with ≤1000 IU vitamin D per day does not alter calcium absorption (8, 14). To clarify the iPTH and fractional calcium absorption response to vitamin D in children, studies using a wide range of inputs and powered to examine race differences are needed.

The IOM report (4) called for dose-response studies in children using >2,000 IU vitamin D per day to help establish an optimal level of 25(OH)D based on functional outcomes. The aims of this study were to determine in children 1) the dose response in 25(OH)D to oral vitamin D_3_; 2) the degree to which vitamin D_3_ supplementation alters fractional calcium absorption, iPTH, and 1,25(OH)_2_D; and 3) whether race and sex modify these responses.

## Subjects and Methods

### Subjects

**Figure 1.**
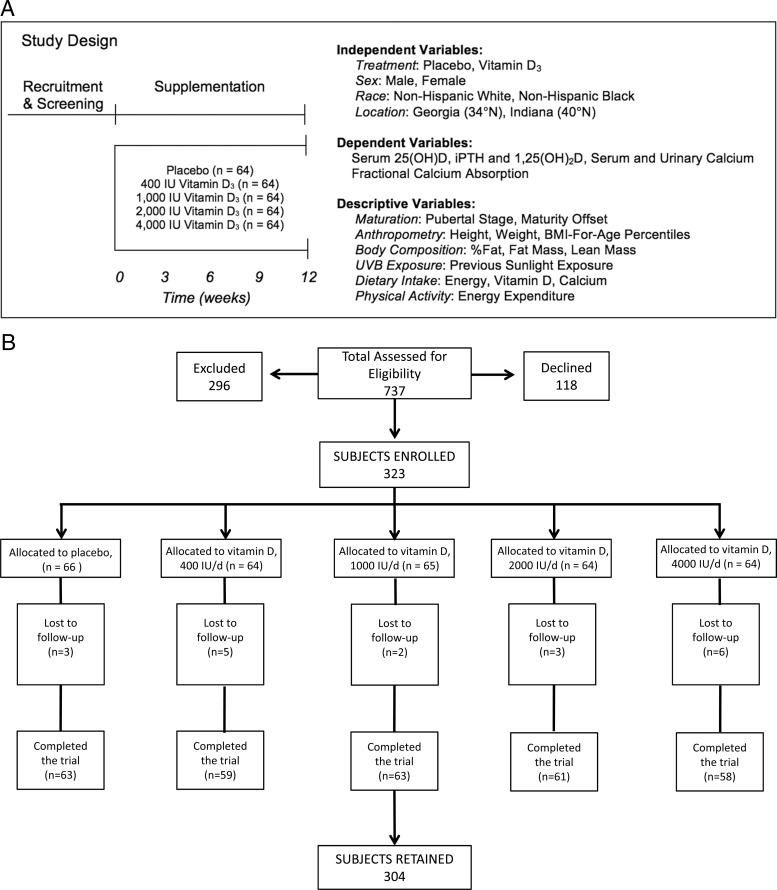
A, Study design. B, Participants recruited and retained.

Males and females aged 9 to 13 years (n = 323) participated in the 12-week randomized, double blind, placebo-controlled GAPI (University of Georgia [UGA], Purdue University [PU], and Indiana University [IU]) trial. Children were enrolled in 2009 to 2010 and 2010 to 2011 during the winter (October through December) when serum 25(OH)D is at its nadir. Study sites included U.S. latitudes 34° N (Athens, Georgia [UGA]) and 40° N (West Lafayette [PU] and Indianapolis [IU], Indiana). Within each of 4 strata defined by race (black/white) and latitude, children were assigned to 1 of 5 vitamin D_3_ doses (Figure 1), with block randomization (blocks were of size 5; the number of treatments) and stratified by sex, race, and latitude. Project statistician (D.B.Hal.) produced the randomization scheme using the blockrand function in R (available at: http://cran.r-project.org/). The supplement manufacturer (Douglas Laboratories) labeled the doses arbitrarily as A, B, C, D, and E. Children were enrolled by 3 study site coordinators, who assigned participants to the intervention based on chronological order of enrollment. Once enrolled, children attended 5 study visits (baseline and weeks 3, 6, 9, and 12). All participants and investigators, including biostatisticians, were blinded to dose until all data were analyzed.

Children were recruited at sexual maturity stages 2 and 3, estimated using self-administered questionnaires for genitalia or breast development (15–18). Both parents and grandparents were the same race as the child and considered themselves non-Hispanic (19). Children taking nutritional supplements were enrolled after a 4-week washout. Children agreed to not alter dietary or physical activity patterns while enrolled. Exclusion criteria included menarche, growth disorders, diseases (eg, cerebral palsy), and medications (eg, corticosteroids) known to influence bone metabolism. Each university's Institutional Review Board for Human Subjects approved the procedures.

### Supplements

Vitamin D_3_ tablets (Douglas Laboratories) contained 0 (placebo), 400, 1000, 2000, or 4000 IU vitamin D_3_. Supplements were confirmed independently (Covance, Inc) as 0.184, 486, 1140, 1880, and 4710 IU vitamin D_3_, respectively. Compliance was estimated by pill counts. A subject was considered compliant if pill bottles were returned at all 5 time points and ≥80% of pills were consumed. Children who returned without pill bottles at ≥1 visit were not included in estimating compliance. A questionnaire was interviewer-administered to seek adverse events information.

### Anthropometric measures

Anthropometric measures included weight (nearest 0.1 kg) using an electronic scale and height (nearest 0.1 cm) using a wall-mounted stadiometer (20). With a one-way random-effects model, single-measure intraclass correlation coefficients (ICCs) were computed among 6- to 10-year-old females (n = 10), measured by the same individual twice over 2 weeks. The ICCs and test-retest coefficients of variation (CV) (percent) were height (0.99% and 0.4%) and weight (0.99% and 1.4%), respectively. Body mass index (BMI)-for-age percentiles were also calculated.

### Biochemical analyses

Fasting blood and second-void urine samples were collected at each visit and stored at <−70°C until analysis. Reference controls (kits) and internal controls (in-house pooled samples) were included with each assay run for quality control. Repeat analyses were conducted when duplicate samples differed by ≥10%. Serum 25(OH)D was assessed using a 2-step RIA (Diasorin) (21, 22). The inter- and intra-assay CV were 5.6% to 8.4% and 5.5% to 7.0%, respectively. Analytical reliability of 25(OH)D assays was further monitored through DEQAS (the Vitamin D External Quality Assessment Scheme). Serum iPTH was measured using an immunoradiometric assay (Diasorin). The inter- and intra-assay CV were 4.8% to 6.9% and 2.3% to 5.7%, respectively. Serum 1,25(OH)_2_D was quantitated using a 2-step RIA (Diasorin). The inter- and intra-assay CV were 12.4% to 17.6% and 11.6%, respectively. Serum and urine calcium (CV = 2.1%) and creatinine (Cr) (CV = 3.5%) were measured using a clinical analyzer (Cobas Mira). Hypercalcemia was defined as serum calcium >10.6 mg/dL and hypercalciuria as urine calcium corrected for Cr >0.22 mg. Serum 25(OH)D values >200 nmol/L characterized hypervitaminosis D.

### Fractional calcium absorption

Fractional calcium absorption was measured at baseline and 12 weeks using a single oral stable isotope and calculated as follows: 1.9458 × (3-hour SSA)^0.8597^ × BSA^1.8608^ × *e*^(−0.1918×Tanner)^, where SSA is serum specific activity expressed as fraction of administered tracer dose per gram of Ca in a 3-hour blood sample; BSA is body surface area, calculated as 0.20247 × (weight [kilograms]^0.425^ × height [meters]^0.725^ (23); *e* is the base of the natural logarithm (∼2.71828), Tanner is pubertal stage. This simplified method, using a single oral isotope and 2 blood samples (3 hours apart), predicts calcium absorption by double-isotope (*R*^2^ = 0.90, *P* < .01) (24). Five milligrams of ^44^Ca as CaCl*_2_* in 1 mL saline was consumed as part of a standardized breakfast containing 150 mg ^40^Ca. Blood was drawn before and 3 hours after isotope administration. Sample preparation was performed by calcium oxalate precipitation, and isotope ratios were measured by High Resolution Inductively Coupled Plasma mass spectrometer.

### Body composition measurement

Fat mass, percent fat, and fat-free soft tissue were assessed at baseline using dual-energy x-ray absorptiometry (DXA) (Delphi-A, Hologic Inc [UGA]; Lunar iDXA, GE Medical Instruments [PU]; and Hologic Discovery-W [IU]). The same technician at each site conducted scans and performed analyses using instrument-specific software and protocols. ICCs were calculated in females aged 5 to 8 years (n = 10) scanned twice at UGA over 7 days for body composition (all ≥0.98). Short- and long-term precision of DXA at IU was <2%. The UGA/PU sites were cross-calibrated by scanning 26 children on the Delphi-A and an iDXA, whereas the IU and PU sites were cross-calibrated by scanning 10 children on the Discovery-W and iDXA. Regression formulae between UGA/PU and IU/PU were derived and used to adjust data from UGA/IU to PU values.

### Demographic, dietary, and physical activity assessment

Parents assisted children in answering interviewer-administered demographic questions and completing 3-day diet records and 3-day physical activity recalls at home on 2 weekdays and 1 weekend day (25–28). Records were analyzed by Food Processor SQL version 9.7.3 (ESHA Research) (29), entered by 2 researchers and statistically compared for agreement. Average measure (3-day) ICCs were calculated in girls aged 6 to 10 years (n = 10), whose 3-day diet records were completed twice over 2 weeks and calculated for vitamin D, calcium, and energy (≥0.86). For the physical activity recalls, a metabolic equivalent (MET) value was assigned to each time block based on the type and intensity of activity described, and average METs per day were calculated.

### Statistical analyses and sample size determination

Statistical analyses were performed using SAS System for Windows version 9.2 (SAS Institute) and R: A Language and Environment for Statistical Computing, version 2.14.1 (R Foundation for Statistical Computing). Descriptive statistics, range, and normality checks and three-way ANOVA with race, sex, latitude, and all interactions at baseline were performed. To investigate the effect of vitamin D supplementation on biochemical outcomes, 25(OH)D and 1,25(OH)_2_D were each modeled over time via a nonlinear mixed-effects model (30) of the following form: *y*_ijkl_ = α_jkl_ + β_jkl_[1 − exp(−*e*^γjkl^*t*_ijkl_)] + ϵ_ijkl_, where *y*_ijkl_ and *t*_ijkl_ denote the measured response (eg, 25[OH]D) and corresponding time (in days from baseline) at measurement on occasion *i* for subject *j* within treatment *k* (*k* = 1, …, 5) of race *l* (*l* = 1, 2). This model is commonly known as the asymptotic regression model or monomolecular growth model and has been used to characterize 25(OH)D levels over time in response to supplementation in previous literature (31). It describes a pattern of change over time in which the response increases smoothly to a long-run or asymptotic level from an initial baseline value. In this model, for the subject corresponding to *j*, *k*, and *l*, α_jkl_ denotes the baseline or initial concentration; α_jkl_ + β_jkl_ denotes the asymptotic concentration, that is, β_jkl_ represents the asymptotic or long-run gain due to supplementation; and γ_jkl_ characterizes the rate of increase from baseline to the asymptote. More specifically, it denotes the natural logarithm of the elimination rate.

Of central interest is the asymptotic gain, β, and how it differs across the supplementation treatments and other experimental factors. Of secondary interest is whether the rate parameter, γ, and, to a lesser extent, baseline value α depend upon experimental factors. To address these questions, each mixed effect was modeled with ANOVA-type specifications involving two-way interactions and main effects among dose and race with additive site and gender effects. For example, the subject-specific long-run gain parameter was specified as β_jkl_ = μ + λ_k_ + τ_l_ + (λτ)_lk_ + δ_1_*M*_jkl_ + δ_2_*G*_jkl_ + *b*_jkl_, where λ_k_, τ_l_ and (λτ)_lk_ are main effects and interactions for treatment and race; *M*_jkl_ and *G*_jkl_ are indicators for male gender and being from Georgia, respectively, with corresponding regression coefficients δ_1_ and δ_2_; and *b*_jkl_ is the normally distributed random subject effect with mean zero. Similar specifications were used for α and γ. These specifications reflect the experimental design and allow a structured analysis of whether the characteristic features of the pattern of change over time in the presence of supplementation depend upon dose (main effect of treatment), whether that dependence has a dose-response form (linear trend in treatment effects), and whether the dose effects differ by race (treatment by race interaction), while controlling for expected differences across sites and gender. These tests were conducted using *F* tests suitable for parametric hypotheses on fixed effects in nonlinear mixed-effects models (30).

As is typical in this class of models, the subject-specific random effects in α, β, and γ were assumed jointly normal and independent across subjects. In addition, the error terms, ϵ_ijkl_, were assumed to be independent, normal random variables with variance proportional to a power of the mean to account for nonconstant (increasing) variance that was observed in residual plots and model diagnostics.

The model was fitted simultaneously to data from all children to yield greater power to determine dose effects and to eliminate problems of nonconvergence encountered by previous researchers who used a 2-stage estimation procedure for a very similar model and application (31). Generally speaking, nonsignificant terms were not removed from the model with the exception of the gender effect in γ, which was found to be nonsignificant in initial model fitting and eliminated to facilitate convergence of subsequent models. This decision was made on practical grounds but is supported by the results of other authors (31) who found the rate parameter to exhibit little variation across subjects.

A test of linear association, on a log-transformed scale as necessary, was conducted to elucidate dependence of iPTH, 1,25(OH)_2_D, and fractional calcium absorption on 25(OH)D. Inverse regression methodology was used to estimate the 25(OH)D value associated with a given targeted mean of 1/iPTH.

Although the primary analysis was intent-to-treat, secondary analyses adjusting for compliance were conducted where β and γ were modeled using analysis of covariance (32). Covariates included significant baseline predictors of subsequent compliance as determined by a separate regression model built for this purpose.

Statistical power was calculated based on fractional calcium absorption, 25(OH)D, 1,25(OH)_2_D, and iPTH with the analysis of 25(OH)D regarded as the primary analysis driving the sample size determination. The sample size necessary to detect a linear change in the asymptotic gain parameter β proportional to dose with a power of 80% was calculated based upon assumptions regarding differences between the 0- and 2000-IU groups within each race via standard sample size and power routines designed for contrasts in multi-way ANOVA models (33). Our primary method of statistical analysis, inference on nonlinear mixed-effects models, can handle arbitrary patterns of missing data and yields valid statistical inferences provided that data are missing at random.

## Results

### Subject characteristics and supplement compliance

Baseline characteristics are shown in Tables 1 and 2. Both blacks vs whites and females vs males were younger and had greater BMI-for-age percentiles. Blacks vs whites had greater lean mass. Males vs females were taller and leaner and had lower fat mass and percent fat. Of those enrolled, 93% were retained (Figure 1B). Approximately 12% (40 of 323) returned without bottles for pill counts at all time points and were not included in the compliance-based subanalyses. Of these, 8 white and 32 black children returned without pill bottles at ≥1 of the time points. Overall compliance was 52.3%, differed across races, with blacks less compliant than whites (*P* = .01), but did not differ across treatments. There was no treatment by race interaction affecting compliance.

**Table 1.** Baseline Characteristics by Race, Sex, and Location^a^

Variable	n	Overall Characteristics							Differences,^b^ *P* < .05
		Overall (n = 323)	White Males (n = 80)	Black Males (n = 82)	White Females (n = 78)	Black Females (n = 83)	Georgia (n = 160)	Indiana (n = 163)	
Age, y	323	11.3 (1.2)	12.1 (1.0)	11.8 (1.2)	11.0 (1.0)	10.5 (1.0)	11.3 (1.2)	11.4 (1.2)	B < W; F < M
Anthropometry									
Weight, kg	323	47.4 (12.2)	47.8 (13.8)	49.4 (13.0)	44.3 (9.3)	47.8 (11.8)	48.0 (11.8)	46.7 (12.5)	
Height, cm	323	151 (9.3)	154 (9.7)	152 (9.6)	149 (8.8)	148 (8.2)	151 (8.9)	151 (9.6)	F < M
BMI-for-age, %	323	68.0 (29.2)	56.6 (32.2)	72.1 (26.1)	66.2 (27.4)	76.7 (27.3)	70.3 (28.7)	65.8 (29.6)	W < B; M < F
Body composition^c^									
Fat mass, kg	320	14.9 (7.4)	13.6 (8.0)	14.0 (7.5)	14.4 (6.1)	17.0 (7.6)	14.2 (7.5)	15.4 (7.3)	M < F
% body fat	320	31.1 (9.4)	28.2 (9.4)	28.4 (9.5)	32.6 (7.9)	35.2 (8.7)	29.7 (9.8)	32.6 (8.7)	M < F
Lean mass, kg	320	30.1 (6.9)	32.2 (7.8)	32.5 (7.6)	28.2 (5.0)	28.6 (5.4)	31.6 (6.9)	29.2 (6.6)	W < B; F < M
Biochemical									
25(OH)D, nmol/L	318	70.0 (18.6)	80.6 (13.4)	61.2 (14.6)	79.6 (16.6)	59.3 (18.0)	72.0 (17.1)	68.0 (19.8)	B < W; IN B < GA B
1,25(OH)_2_D, pmol/L	318	144 (42.8)	130 (34.6)	146 (46.4)	147 (47.9)	152 (38.6)	146 (45.6)	142 (39.9)	W < B; M < F
iPTH (pg/mL)	318	27.4 (10.8)	24.2 (9.0)	28.4 (11.3)	25.7 (9.2)	31.1 (12.1)	28.7 (11.4)	26.1 (10.0)	W < B
Fractional Ca absorption	311	0.44 (0.14)	0.46 (0.14)	0.39 (0.14)	0.47 (0.13)	0.45 (0.13)	0.43 (0.14)	0.45 (0.13)	B < W; M < F
Urine Ca (Ca/Cr)	305	0.06 (0.08)	0.07 (0.06)	0.05 (0.05)	0.08 (0.08)	0.05 (0.12)	0.06 (0.09)	0.07 (0.07)	B < W
Serum Ca, mg/dL	305	9.8 (0.3)	9.7 (0.3)	9.9 (0.3)	9.9 (0.4)	9.9 (0.3)	9.8 (0.3)	9.9 (0.4)	W < B; WM < WF; GA < IN
Dietary intake (per day)									
Energy, kcal	307	2001 (556)	2143 (541)	2025 (602)	1975 (543)	1853 (505)	1944 (528)	2053 (577)	F < M
Vitamin D, IU	307	169 (124)	188 (163)	168 (95)	157 (121)	162 (105)	153 (101)	184 (142)	GA < IN
Calcium (mg)	307	901 (395)	964 (417)	849 (401)	964 (419)	825 (321)	888 (373)	912 (416)	B < W; GA M < IN M
Energy expenditure, METs/d	304	62.2 (10.0)	63.4 (9.0)	61.3 (11.3)	64.8 (10.2)	59.3 (8.7)	63.4 (10.5)	61.1 (9.4)	B < W; IN B < GA B

Abbreviations: B, black; F, female; GA, Georgia; IN, Indiana; M, male; W, white.

a Values are presented as means (SD). Overall characteristics represent data collapsed across the 5 treatment groups.

b Results of three-way ANOVA for race, gender, latitude, and all interactions investigated. Differences shown are significant at α = .05.

c Body composition measures assessed using DXA.

**Table 2.** Baseline Characteristics by Treatment^a^

Variable	n	Characteristics by Vitamin D Dose				
		Placebo (n = 66)	400 IU (n = 64)	1000 IU (n = 65)	2000 IU (n = 64)	4000 IU (n = 64)
Age, y	323	11.5 (1.2)	11.3 (1.2)	11.1 (1.1)	11.4 (1.4)	11.5 (1.2)
Anthropometry						
Weight, kg	323	45.5 (11.3)	46.6 (10.4)	46.1 (11.1)	52.0 (14.8)	46.7 (12.2)
Height, cm	323	151 (8.9)	151 (8.1)	149 (9.2)	153 (10.4)	150 (9.4)
BMI-for-age, %	323	63.3 (29.5)	67.6 (27.8)	70.4 (28.4)	71.5 (30.4)	67.4 (30.1)
Body composition^b^						
Fat mass, kg	320	13.7 (7.0)	14.3 (7.1)	14.6 (7.3)	16.3 (8.2)	14.9 (7.5)
% body fat	320	29.8 (8.9)	30.8 (9.4)	31.5 (9.9)	31.6 (9.7)	31.9 (9.0)
Lean mass, kg	320	30.0 (6.3)	30.1 (5.6)	29.2 (5.8)	32.9 (9.1)	29.7 (6.6)
Biochemical^3^						
25(OH)D, nmol/L	318	71.5 (18.6)	71.4 (19.5)	71.1 (19.7)	65.8 (7.3)	70.0 (17.5)
1,25(OH)_2_D, pmol/L	318	146 (45)	143 (40)	140 (39)	149 (44)	142 (46)
iPTH, pg/mL	318	26.6 (10.8)	27.6 (9.1)	26.2 (10.7)	30.2 (12.8)	26.4 (9.9)
Serum Ca, mg/dL	305	9.8 (0.3)	9.9 (0.3)	9.9 (0.3)	9.9 (0.3)	9.8 (0.3)
Urine Ca (Ca/Cr)	305	0.07 (0.06)	0.07 (0.13)	0.08 (0.10)	0.05 (0.05)	0.05 (0.05)
Fractional Ca absorption	310	0.46 (0.16)	0.45 (0.15)	0.44 (0.20)	0.47 (0.17)	0.46 (0.17)
Dietary intake (per day)						
Energy, kcal	307	1978 (513)	1996 (577)	1986 (523)	2000 (642)	2048 (536)
Vitamin D, IU	307	151 (96)	198 (140)	143 (111)	184 (160)	175 (101)
Calcium, mg	307	837 (321)	1000 (467)	822 (375)	914 (411)	945 (378)
Energy expenditure, METs/d	304	60.9 (8.2)	63.8 (12.0)	61.0 (10.0)	63.0 (9.2)	62.4 (10.3)

a Values are presented as means (SD).

b Body composition measures assessed using DXA.

### Serum 25(OH)D

**Figure 2.**
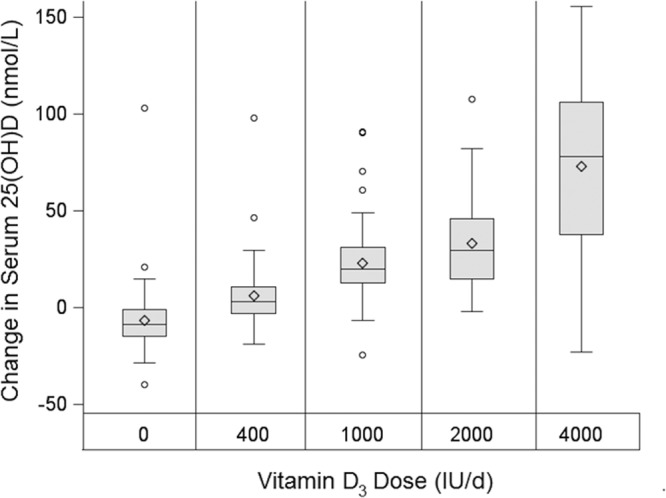
Change in serum 25(OH)D after 12 weeks of D3 supplementation (n = 323). In the schematic box plots, diamonds indicate means, horizontal lines indicate medians, shaded boxes indicate interquartile ranges (IQR), whiskers indicate highest value below the upper fence (1.5 × IQR above the 75th percentile) and the lowest value above the lower fence (1.5 × IQR below the 25th percentile), and circles indicate values outside the upper and lower fences. For vitamin D dose, *P* < .0001 for trend.

**Figure 3.**
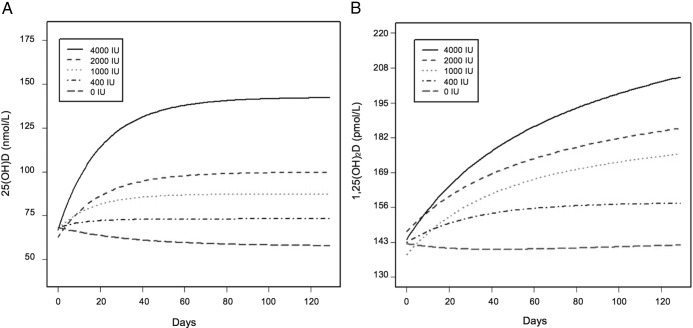
A, Fitted 25(OH)D curves over time for the overall sample (n = 323). B, Fitted 1,25(OH)_2_D curves over time for the overall sample (n = 323). The majority of subjects completed the study within 12 weeks; however, data were included from several subjects who were enrolled up to 65 days after the 12-week intervention.

Serum 25(OH)D ranged from 25.3 to 114.7 nmol/L at baseline where 15% (47 of 318) of children had values <50 nmol/L (insufficiency), 6% (18 of 318) had values <40 nmol/L (estimated average requirement), and <1% (3 of 323) of children had values <30 nmol/L (deficiency). After 12 weeks, 25(OH)D ranged from 24.2 to 237.4 nmol/L with 9% (26 of 302) having serum 25(OH)D <50 nmol/L. At baseline, blacks vs whites had lower 25(OH)D and blacks living in Indiana vs Georgia had lower 25(OH)D. Changes in serum 25(OH)D in response to supplementation are illustrated in Figure 2. Serum 25(OH)D increased in a dose-dependent manner, and higher doses resulted in higher long-run concentrations (Figure 3A; *P* < .01). The rate of increase (γ) in 25(OH)D was not different by dose or race. The mean values for the equation parameters predicting serum 25(OH)D by treatment are shown in Table 3. The mean increment in 25(OH)D increased with dose and changes over 12 weeks ranged from −10 nmol/L for placebo to 76 nmol/L for the 4000-IU dose. The increment with each dose was significantly different from placebo (*P* < .05), except for the 400-IU dose in whites. To obtain prediction equations for β, a reduced model was fit in which qualitative effects of dose were replaced by the statistically significant quantitative dose effects (linear in the case of whites, linear and quadratic for blacks). This model was validated, found to fit similarly to the original model (likelihood ratio test statistic = 5.99 on 5 degrees of freedom, *P* = .307) and yielded the following population-level prediction equations for the asymptotic gain: blacks, a_i_ = −8.01 + 5.22latitude_i_ − 3.99sex_i_ = 3.27dose_i_ − 0.417dose^2^; whites, a_i_ = −9.73 + 5.22latitude*_i_* − 3.99sex_i_ = 2.55dose_i_, where latitude_i_ = 1 if subject was from 34° N, 0 if 40° N; sex_i_ = 1 if subject was male, 0 if female; and dose_i_ = dose in hundreds of international units.

**Table 3.** Model Variables to Predict 25(OH)D by Daily Vitamin D_3_ Dose^a^

Dose, IU/d	Parameter					
	*C*(0), nmol/L		*a*, nmol/L		*k*	
	Estimate	SE	Estimate^b^	SE	Estimate	SE
0	70.89	1.90	−10.12	2.86	−3.67	0.32
400	70.82	1.93	5.54	2.59	−2.90	0.52
1,000	70.22	1.92	20.29	2.61	−2.91	0.17
2,000	65.35	1.92	37.57	2.66	−3.08	0.10
4,000	69.54	1.94	76.07	2.95	−3.05	0.07

a Means are ± SE in nanomoles per liter by daily vitamin D_3_ dose, averaged over race, sex, and latitude. *C*(*t*) = *C*(0) + *a*[1 − exp(−exp(*k*)*t*)] + *e*(*t*), where, *C*(*t*) is the concentration of 25(OH)D at time *t* for each subject, *C*(0) is the corresponding initial concentration of 25(OH)D at baseline, *C*(0) + *a* is the asymptotic or equilibrium concentration as *t*→∞ for a given constant supplementation level, *k* is the natural log elimination rate constant, and *e*(*t*) is a mean zero error term.

b The increment with each dose was significantly different from placebo (*P* < .05); the 400-IU dose increment for whites was not different from placebo.

Pairwise comparisons using Bonferroni adjustments between each of the 4 treatments and placebo showed that 25(OH)D gains in each dose were different from placebo (*P* < .01), except for 400 IU in whites. Table 4 shows that larger 25(OH)D gains were observed for whites vs blacks at the highest dose (*P* < .01). To determine whether adiposity modified the treatment effect on the asymptotic gain in 25(OH)D, the original model was refit with baseline fat mass as a covariate in the submodels for parameters α, β, and γ. Results from this secondary analysis showed that the dose effects on 25(OH)D were not modified while taking fat mass into consideration.

**Table 4.** Model Variables to Predict 25(OH)D by Race in the 4000-IU Group^a^

Race	Parameter: *C*(0) + *a*, nmol/L	
	Estimate	SE
Black	117.21	4.43
White	174.03	5.15
Averaged over race	145.62	3.40

Means are ± SE in nanomoles per liter for the 4000-IU group, averaged over sex and latitude. Larger 25(OH)D gains were observed for whites vs blacks at the 4000-IU dose (*P* < .01). See Table 3 for explanation of terms.

### Serum 1,25(OH)_2_D and iPTH

Both blacks vs whites and females vs males had higher 1,25(OH)_2_D at baseline. No differences in 1,25(OH)_2_D were observed by latitude. Figure 3B illustrates the 12-week time course of 1,25(OH)_2_D by dose for the overall sample. Asymptotic increases for 1,25(OH)_2_D were not statistically significant (*P* = .07). There was also no evidence of race differences in the increase from baseline to asymptote. At baseline, blacks vs whites had higher iPTH. Despite the fact that 25(OH)D and iPTH were inversely correlated (*r* = −0.37, *P* < .01), iPTH did not change in a dose-dependent manner with supplementation, nor were there differences between races from baseline to asymptote.

### Fractional calcium absorption

**Figure 4.**
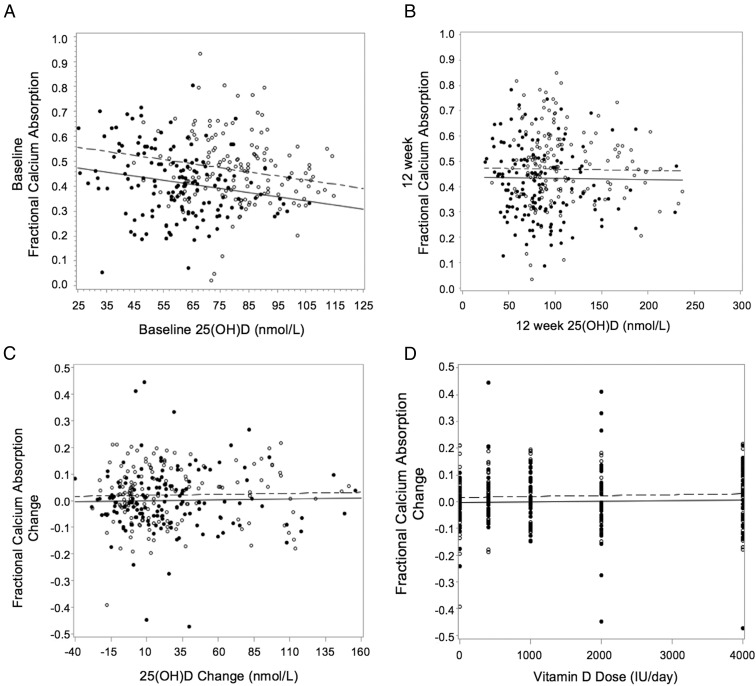
A–C, Relationship between serum 25(OH)D and fractional calcium absorption at baseline (A) (serum 25(OH)D, *P* = .001; race [white vs black], *P* < .0001; slope = −0.002, *R*^2^ = 0.073, n = 297); 12 weeks (B) (25(OH)D, *P* = .13; race (white vs black), *P* < .0001; *R*^2^ = 0.071, n = 297) and (C) as change from baseline to 12 weeks (C) (25(OH)D, *P* = .66; race, *P* = .12; *R*^2^ = 0.013, n = 297). D, Relationship between vitamin D dose and change in fractional calcium absorption after 12 weeks of supplementation (vitamin D dose, *P* = .54; race, *P* = .12). Filled circles and solid lines indicate blacks, and open circles and dotted lines indicate whites.

Baseline fractional calcium absorption was lower in blacks vs whites and in males vs females. At baseline, 25(OH)D and fractional calcium absorption were not related; however, when adjusted for race, a negative relationship existed between 25(OH)D with higher absorption for whites than blacks (Figure 4A). The relationship between 25(OH)D and fractional calcium absorption did not differ by sex or latitude. There was no relationship between 12-week 25(OH)D and fractional calcium absorption before or after adjusting for race (Figure 4B). There was also no effect of vitamin D on fractional calcium absorption (change from baseline to 12 weeks) (Figure 4D). Change in 25(OH)D was not related to change in fractional calcium absorption before or after adjusting for race (Figure 4C) and was not affected when adjusted for sex, latitude, baseline 25(OH)D, or compliance. Furthermore, 1,25(OH)_2_D was not related to fractional calcium absorption at baseline or 12 weeks, and taking race, sex, and latitude into account did not affect these relationships.

### Supplement safety

Black vs white children had lower baseline urinary calcium and higher serum calcium. No differences in baseline urinary calcium were observed by sex or latitude. Among whites only, females vs males had higher serum calcium. Children living in Indiana vs Georgia had higher serum calcium. Over 12 weeks, 3 children met the criteria defining hypercalciuria, 3 for hypercalcemia, and 7 for hypervitaminosis D.

### Diet and physical activity

Males had greater energy intake vs females at baseline. Children living in Indiana vs Georgia had greater intake of vitamin D. Both whites vs blacks and males living in Indiana vs Georgia consumed more calcium. Whites vs blacks and blacks living in Georgia vs Indiana had greater energy expenditure.

## Discussion

The IOM described difficulties in establishing pediatric Dietary Reference Intakes for vitamin D partially because of the lack of dose-response studies (4). Many of the studies used to simulate these dose-response data included 1 lower dose or placebo and 1 single, larger dose. The GAPI trial was the first multisite, randomized dose-response trial conducted in children with vitamin D_3_ doses ranging from 400 to 4000 IU/d. Higher doses resulted in higher asymptotic 25(OH)D responses in both white and black children, but there was no effect on iPTH or calcium absorption. Although not statistically significant, 1,25(OH)_2_D exhibited a roughly asymptotic increase over time.

The dose-dependent increases in 25(OH)D in this study were lower than reported in other trials using a range of 200 to 2000 IU/d (6, 9) and higher than observed in a study using either 200 or 1000 IU/d (34). The response to supplementation with 2000 IU/d in the current study was approximately 38 nmol/L, lower than 60 nmol/L observed when ∼2000 IU/d was provided to U.S. black adolescents over 16 weeks (6) or to female Lebanese adolescents over 1 year (9). The difference in serum responses is likely due to higher baseline 25(OH)D. Even with constrained wintertime testing to minimize UVB exposure, baseline 25(OH)D was ∼70 nmol/L in this study, more than double baseline concentrations observed in previous trials. The IOM recommends intakes of 600 IU/d for children (4), but there is considerable debate as to whether this recommendation is sufficient. Although the present trial was conducted for only 12 weeks, we found among healthy children with 25(OH)D ∼70 nmol/L that vitamin D_3_ supplementation with 400 IU/d was sufficient in maintaining wintertime 25(OH)D in healthy black, but not white, children.

An important goal of this project was to ascertain whether race modified the biochemical responses of vitamin D metabolism with increasing vitamin D doses. There was a significant interaction between race and dose, such that at 400, 1000, and 2000 IU/d, there were equal 25(OH)D gains for whites and blacks, but at 4000 IU, whites had greater gains than blacks. Other trials that included black adolescents (5, 6) and one with adults (35, 36) demonstrated that 25(OH)D responses were similar among races. Rather than a biological explanation for the race interaction in this study, it is likely that compliance contributed to the discrepancy observed at 4000 IU. However, because our estimation of compliance included only children who returned pill bottles at all visits, we were not able to capture a significant dose by race interaction in compliance rates. It is noteworthy that 32 black vs 8 white children did not return pill bottles at ≥1 testing occasion. Of these, 6 of 32 black children and 2 of 8 white children were assigned 4000 IU/d.

This study was one of the few to assess 1,25(OH)_2_D responses to vitamin D supplementation. Unlike findings in children supplemented with ≤1000 IU vitamin D per day (7, 37), we report that there was a marginal, albeit nonsignificant, increase in 1,25(OH)_2_D with increasing dose, which was not different by race. These results are consistent with studies that used 1600 or 2000 IU/d regardless of baseline 25(OH)D. The marginal increases in serum 1,25(OH)_2_D observed in the present study should have been linked with increased calcium absorption, but that was not the case. Assessment of serum 1,25(OH)_2_D using liquid chromatography–mass spectrometry should be pursued to better characterize the 1,25(OH)_2_D increases observed in this study.

Based on adult data (35), it would be expected that increases in 25(OH)D and 1,25(OH)_2_D would be accompanied by iPTH suppression after supplementation. Cross-sectional studies in children have reported inverse or no association between iPTH and 25(OH)D, and in one trial in younger children, a significant reduction in iPTH occurred after 13 weeks of supplementation with 1000 IU/d (8). We report that 25(OH)D and iPTH were inversely correlated at baseline. However, after 12 weeks of supplementation with doses up to 4000 IU/d, there was no effect of increased 25(OH)D on iPTH suppression. In adults, the inflection point at which maximal suppression occurs is used in defining vitamin D requirements. Based on the findings from the current trial, the use of iPTH suppression in children for identifying recommended 25(OH)D cutoffs is not warranted.

The most relevant functional outcome in this trial, fractional calcium absorption, did not improve over 12 weeks with increased 25(OH)D. This confirms cross-sectional (38) and intervention data in children (8, 14) who received 1000 IU/d. The null effect of supplementation on fractional calcium absorption implies that baseline 25(OH)D, ranging from 25.3 to 114.7 nmol/L, can be considered adequate for fractional calcium absorption in this age during winter. The response of fractional calcium absorption to vitamin D supplementation was not affected by baseline 25(OH)D; however, 85% of children had concentrations >50 nmol/L throughout the study. Therefore, our findings may not be generalizable to children with lower 25(OH)D. This study also showed that, despite lower baseline calcium absorption and 25(OH)D in blacks vs whites, concomitant increases in 25(OH)D and 1,25(OH)_2_D with supplementation did not result in calcium absorption increases. Therefore, 25(OH)D in healthy black children at baseline was adequate for calcium absorption.

This pediatric trial was the first to employ a daily dose >2000 IU/d; therefore, it was important to monitor safety particularly in the group receiving 4000 IU. Serum calcium indicative of hypercalcemia occurred in 3 children, and another 3 distinct children had urinary calcium levels defined as hypercalcuria. None of these children were assigned to 4000 IU/d, and it did not appear that these elevated levels were associated with dose. In contrast, 7 children, or 10% of those receiving 4000 IU/d met the criteria for hypervitaminosis D by the trial's end. The consequences of these high serum levels are unknown, but because no subject reported adverse events over the 12-week study, we conclude that short-term supplementation with 4000 IU/d appears safe. Longer-term studies are needed to ascertain the safety of 25(OH)D exceeding 200 nmol/L. In conclusion, vitamin D_3_ doses ≤4000 IU/d appear to be safe over the short term and indicate that healthy U.S. children do not require vitamin D supplementation to improve calcium absorption.
